# Greater income and financial well-being are associated with higher prosocial preferences and behaviors across 76 countries

**DOI:** 10.1093/pnasnexus/pgae582

**Published:** 2025-02-04

**Authors:** Paul Vanags, Jo Cutler, Fabian Kosse, Patricia L Lockwood

**Affiliations:** School of Psychology, Centre for Human Brain Health, University of Birmingham, Birmingham BT15 2TT, United Kingdom; School of Psychology, Centre for Developmental Science, University of Birmingham, Birmingham BT15 2TT, United Kingdom; School of Psychology, Institute for Mental Health, University of Birmingham, Birmingham BT15 2TT, United Kingdom; School of Psychology, Centre for Human Brain Health, University of Birmingham, Birmingham BT15 2TT, United Kingdom; School of Psychology, Centre for Developmental Science, University of Birmingham, Birmingham BT15 2TT, United Kingdom; School of Psychology, Institute for Mental Health, University of Birmingham, Birmingham BT15 2TT, United Kingdom; Department of Economics, Julius-Maximilians-Universität Würzburg, Sanderring 2, Würzburg 97070, Germany; School of Psychology, Centre for Human Brain Health, University of Birmingham, Birmingham BT15 2TT, United Kingdom; School of Psychology, Centre for Developmental Science, University of Birmingham, Birmingham BT15 2TT, United Kingdom; School of Psychology, Institute for Mental Health, University of Birmingham, Birmingham BT15 2TT, United Kingdom

**Keywords:** prosocial, wealth, altruism, trust, reciprocity

## Abstract

Prosocial preferences and behaviors—defined as those that benefit others—are essential for health, well-being, and a society that can effectively respond to global challenges. Identifying factors that may increase or decrease them is therefore critical. Wealth, in the form of income or subjective financial well-being (FWB), may be crucial in determining prosociality. In addition, individuals’ experience of precarity (inability to meet basic needs) or country-specific factors could change how wealth correlates with prosociality, yet this impact is unknown. Here, we tested how self-reported household income (HHI) and FWB were associated with seven measures of prosociality in a global, representative sample of 80,337 people across 76 countries. We show a consistent positive association between wealth and prosociality, across both measures and for both financial and nonfinancial prosocial preferences and behaviors. HHI was positively associated with altruism, positive reciprocity, donating money, volunteering, and helping a stranger, but negatively associated with trust. FWB was positively associated with all aspects of prosociality, including trust. Individuals’ experience of precarity reduced the strength of wealth associations for prosocial preferences but increased them for prosocial behaviors. Positive associations between wealth and prosociality were found around the world and across country-level wealth and cultural factors. These findings could have important implications for enhancing prosociality, critical for a healthy and adaptive society.

**Significance Statement**

Prosocial behaviors that help others are essential for addressing global challenges and an adaptive society. These behaviors may be linked to income levels, as well as subjective feelings about one's overall financial situation. We show in a globally representative sample of 80,337 people across 76 countries that higher wealth is associated with multiple indexes of self-reported prosocial preferences and behaviors. We also found that the strength of these associations changed dependent on whether people had experienced precarity in the past, whether they were in a collectivist culture, and how wealthy their country was. Our results suggest future avenues for increasing prosociality that could be relevant across the globe.

## Introduction

Human beings show remarkable levels of prosociality—preferences and behaviors that benefit others (1, 2). Prosociality has been essential to the evolutionary success of our species (3, 4) and remains an important source of physical and mental well-being (5,6). Understanding the multiple facets of prosociality, and how to promote helping others, is therefore vital and a focus of several research fields. One critical factor is wealth, both how much money someone earns through income and how they subjectively feel about their financial situation. However, previous research tends to consider single aspects of wealth and single measures of prosociality. In addition, existing work has often focused on testing people who are predominantly from Western, industrialized, rich, educated, and democratic countries (7). It is critical that our understanding of preferences and behaviors is representative around the world, particularly for financial factors that vary dramatically outside the countries usually studied (8). Here, we examined how two aspects of wealth, self-reported household income (HHI) and financial well-being (FWB), are associated with seven survey assessments of prosocial preferences and behaviors in the global population. While these measures of wealth are correlated, they do not overlap completely and may show different associations with prosocial behavior.

To date, the majority of studies that measure the association between income and prosociality find a positive relationship (9–14). Country-level incomes as measured by gross domestic product have also been shown to correlate with real-world prosocial behaviors such as returning lost wallets (15). However, one study showed that lower family incomes were associated with a belief that people should donate more (16) and there is some evidence for a “U”-shaped profile to charitable giving with lowest and highest income households donating more (17). As well as previous data suggesting the importance of associations between prosociality and wealth, theoretical accounts point to particular hypotheses. For example, models of inequity aversion imply a positive association between wealth and prosociality via mechanisms of income redistribution (18, 19).

Few, if any, studies have examined the association between subjective FWB and prosociality specifically, as these are typically bound up in measures of subjective socioeconomic status (SES). SES is a broad concept generally measured as a composite of income, educational attainment, and occupational status, with inconsistency between studies (20). Several studies have suggested that, in contrast, higher subjective SES is associated with lower levels of prosocial behavior (16, 21–25). However, others have questioned these results (20, 26–30).

In addition to the variety of measures used, a possible explanation for these mixed findings is that the association between wealth and prosociality is moderated by additional factors. One such factor is the experience of precarity, defined here as an inability to meet basic needs such as food and shelter. Research suggests important psychological and behavioral consequences of precarity (31–33). Theoretically, precarity may reduce prosocial behavior (34). However, a meta-review examining precarity and prosocial behavior showed physiological scarcity (hunger/thirst) increased prosocial behaviors, whereas financial scarcity did not (35). Another study showed that precarity in childhood was associated with lower volunteering (36). Experience of precarity may also moderate how wealth is associated with prosociality. Precarity could increase the desire to help, due to mechanisms of empathy and shared experience, such as the empathy–altruism hypothesis (37). Alternatively, the association with wealth may be smaller or not evident within people who have experienced precarity if this limits their ability to help, regardless of income or FWB.

Here, we conducted a preregistered analysis of data from 80,337 people from 76 countries, representing 90% of the global population. We quantified the correlations of income (HHI in international dollars, adjusted to each country) and FWB with seven measures of self-reported prosociality. Four of these measured prosocial preferences (positive reciprocity, altruism, trust, and negative reciprocity) and three captured real-world behaviors (donating money, volunteering, and helping a stranger). Crucially, these also covered financial measures, those that referenced monetary giving, and nonfinancial measures. We controlled for several factors that could covary with wealth or prosociality, including gender, age, cognitive ability, and physical health. We also tested the moderation of precarity, indexed by self-reported experience of inability to provide food/shelter in the previous year. Finally, we considered consistency across countries and whether country-level gross national income (GNI) and cultural factors moderated wealth–prosociality associations.

Based on previous empirical findings, our preregistered hypotheses were that higher income would be associated with greater levels of prosociality (9–14), whereas FWB would show the opposite (16, 21–25). We additionally tested whether correlations varied based on the type of prosocial preference or behavior. Specifically, we predicted that all forms of prosociality would have a positive relationship with income, but the effect size would be greater for financial measures that involved monetary amounts (donations, altruism, positive reciprocity) than nonfinancial forms of prosociality (volunteering, helping, trust, negative reciprocity).

We find that both income and FWB are positively associated with multiple prosocial preferences and behaviors globally. In general, people who reported higher income or FWB are more likely to report helping others. An exception was trust, which showed a positive association with FWB but a negative association with income. As predicted, stronger wealth relationships were found for financial measures but crucially, higher wealth had a positive association with prosociality on nonfinancial measures. We additionally show that precarity influences associations between wealth and prosociality enhancing the strength of associations for prosocial behaviors and reducing them for prosocial preferences. The positive associations of wealth with positive reciprocity, altruism, donating money, volunteering, and helping a stranger were also highly consistent across countries. While country-level GNI and how individualistic or collectivist the country is moderated some correlations, higher wealth was robustly associated with increased prosociality across different levels of these factors. Together, the unique combination of measures provides a powerful way of assessing how wealth relates to prosociality and shows decisive evidence for a positive association.

## Results

We analyzed the link between wealth and prosociality in two linked, globally representative datasets, the Global Preferences Survey (GPS) and the Gallup World Poll (GWP). These cover 80,337 individuals, representative samples from 76 countries around the world. We fit (generalized) linear mixed-effects models (LMMs) for each combination of wealth measure and prosociality variable (see Methods). Each model contained a wealth measure and controls for gender, age, cognitive ability, and physical health. As in previous studies (9), HHI was log-transformed, and our analysis focused on linear associations (see Table S1 for quadratic models). We report significance at *P* < 0.01 in all analyses using the whole dataset (note that this deviated from our preregistered *P* < 0.05 to increase robustness, given the large sample).

### Greater income and FWB are associated with increased prosocial preferences and behaviors all over the world

**Fig. 1.**
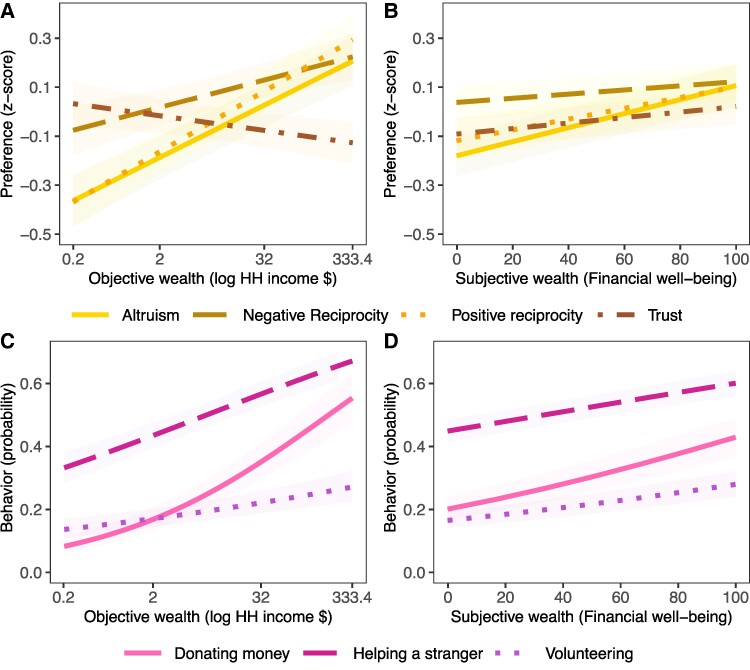
Greater wealth is associated with higher prosociality around the world. A) Income showed positive associations with altruism, positive reciprocity, and negative reciprocity and a negative association with trust, B) FWB was positively associated with all preferences, C) Income was positively associated with all behaviors with donating being particularly strong, and D) FWB was positively associated with all behaviors. Plots show predictions from LMMs of prosocial preferences/behaviors, controlling for gender, age, physical health, and cognitive ability. Preferences were modeled as standardized continuous variables, and binary behaviors were modeled with generalized LMMs (see Methods). Linear models are shown here with the quadratic models reported in supplementary materials. Plots were created by predicting response data from model fits, the shaded area representing 95%CI (plots showing individual data points, Figs. S1–S4).

Income was positively associated with altruism (standardized β [95% CI] = 0.097 [0.077 0.117], *P <* 0.001), negative reciprocity (β=0.051 [0.027 0.075], *P <* 0.001), and positive reciprocity (β=0.112 [0.089 0.136], *P <* 0.001) (Fig. 1A–D, Table S2). In other words, people with higher incomes were more likely to claim giving money to good causes and report returning prosocial behavior toward them, while punishing unfair behavior. However, contrary to our hypothesis, a negative association between income and trust showed those with higher incomes reported lower trust in others (β=−0.027 [−0.046 −0.008], *P =* 0.005). Income was also positively associated with all measures of reported prosocial behavior: donating (odds ratio [OR] = 1.562 [1.443 1.690], *P <* 0.001), volunteering (OR = 1.157 [1.099 1.218], *P <* 0.001), and helping a stranger (OR = 1.271 [1.210 1.336], *P <* 0.001; Fig. 1A–D, Table S2). Therefore, greater income was associated with increased prosociality across a broad range of preferences and behaviors, except for trust.

To examine whether the negative association between income and trust was related to inclusion of country-level random effects, not present in previous studies, we re-ran our analysis using a simple linear regression model without random effects. Results showed a positive association between income and trust (β=0.038 [0.029 0.046], *P <* 0.001) when country-level influences were not accounted for. This suggests that failing to account for differences between countries in average levels of trust and how wealth is associated with trust can obscure or reverse the negative relationship between wealth and trust at an individual level.

Next, we examined FWB. Contrary to our preregistered hypotheses, FWB was positively associated with increased prosociality on all measures. Individuals who subjectively reported greater FWB were more prosocial, for both preferences (altruism: β=0.085 [0.072 0.098], *P <* 0.001; trust: β=0.033 [0.018 0.049], *P <* 0.001; negative reciprocity: β=0.025 [0.009 0.041], *P =* 0.002; positive reciprocity: β=0.065 [0.050 0.081], *P <* 0.001) and behaviors (donating: OR = 1.384 [1.334 1.436], *P <* 0.001; volunteering: OR = 1.221[1.177 1.267], *P <* 0.001; helping a stranger: OR = 1.200 [1.153 1.250], *P <* 0.001). Therefore, both higher incomes and FWB were associated with increased prosocial behaviors and most preferences. Interestingly, income and FWB showed opposing associations with trust. People with higher incomes reported being less willing to trust others, whereas those who reported higher FWB showed greater levels of trust.

### Wealth has stronger positive associations with financial prosociality than nonfinancial measures

Having observed significant wealth associations with all measures of prosocial preferences and behaviors, we next compared the size of these associations between measures. We hypothesized that wealth would be more strongly associated with financial measures involving monetary amounts than nonfinancial prosociality. We compared the absolute size of regression coefficients within each set of models using *z*-scores based on pooled variance (38). The association with income was significantly larger for the financial preferences altruism (A) and positive reciprocity (*P*) than nonfinancial trust (T) and negative reciprocity (N) (*Z*_A–T_ = 6.89, *P <* 0.001; *Z*_P–T_ = 6.76, *P <* 0.001; *Z*_A–N_ = 4.07, *P <* 0.001; *Z*_P–N_ = 4.53, *P <* 0.001; Table S3 for all comparisons). Similarly, FWB had significantly larger correlations with altruism and positive reciprocity compared with negative reciprocity and trust (*Z*_A–T_ = 6.90, *P <* 0.001; *Z*_P–T_ = 4.93, *P <* 0.001; *Z*_A–N_ = 8.26, *P <* 0.001; *Z*_P–N_ = 6.48, *P <* 0.001).

For the associations of wealth on the (binary) prosocial behaviors, we compared financial donating to nonfinancial helping and volunteering, again using *z*-scores capturing the difference between ORs. As hypothesized, income had a larger correlation with donating than helping and volunteering (*Z*_D–V_ = 3.24, *P* < 0.001; Z_D–H_ = 2.11, *P =* 0.017). FWB also showed the largest correlation with donating, compared with helping and volunteering (*Z*_D–H_ = 2.75, *P =* 0.003; *Z*_D–V_ = 2.38, *P =* 0.009). To summarize, all measures displayed significant associations with income and FWB, and as predicted, the most positive associations of wealth were on financial measures, altruism, positive reciprocity, and donating.

### Precarity moderates associations between wealth and prosociality

Next, we examined how precarity moderated the significant associations between wealth and prosociality. Here, we assessed precarity as inability to access food and shelter in the past 12 months (GWP Food and Shelter Index, see Supplementary Methods). Summing participants’ binary responses to each question (food and shelter) created four levels (no precarity, food precarity, shelter precarity, or both). Precarity is distinct from FWB as precarity questions did not ask how satisfied participants were with their situation, only whether they could meet these basic needs, and whether the two measures were only weakly correlated (see Methods).

**Fig. 2.**
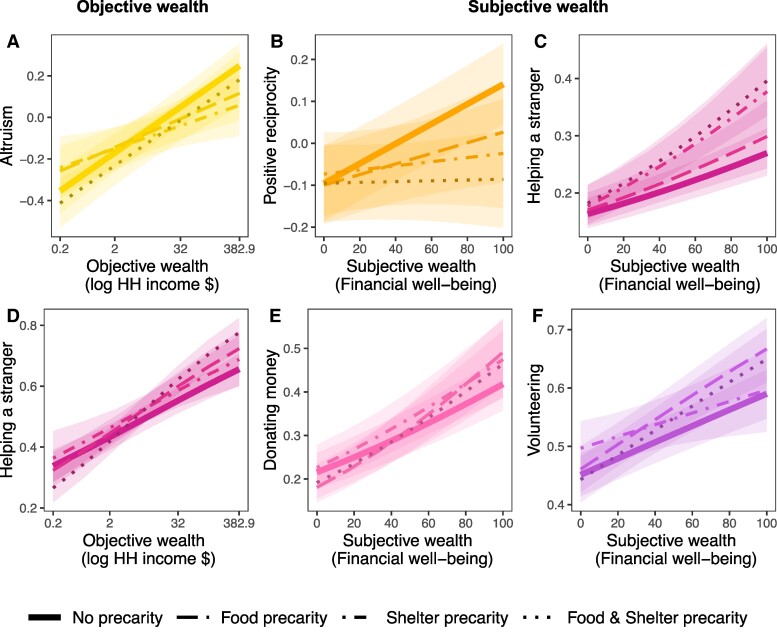
Experience of precarity reduced associations between wealth and prosocial preferences but enhanced associations with prosocial behaviors. Models tested an interaction effect between wealth and a four-level precarity factor (no precarity, food precarity, shelter precarity, and both), with the significant interactions plotted above. The two significant interactions for preferences, A) income–altruism and B) FWB and positive reciprocity, were both negative, meaning that experience of precarity decreased the strength of the wealth–prosociality association. C) In contrast, the positive interaction between precarity and the income—helping a stranger relationship meant the strongest association with wealth was for those with experience of precarity. All three interactions between FWB and precarity with D) helping a stranger, E) donating, and F) volunteering were also positive.

We found that the experience of precarity reduced the positive association between income and altruism (*Χ*^2^ = 15.1, *P =* 0.002) and the positive association between FWB and positive reciprocity (*Χ*^2^ = 31.5, *P <* 0.001). Those that had experienced precarity had a less pronounced increase in these prosocial preferences with wealth than those that had not (Fig. 2A and B, Table S4). Interestingly, the moderation on behaviors acted in the opposite direction (Fig. 2C–F). Precarity increased the positive associations of FWB on donating and volunteering (*Χ*^2^ = 19.0, *P <* 0.001; *Χ*^2^ = 15.4, *P =* 0.002) and the positive associations of both income and FWB on helping a stranger (*Χ*^2^ = 21.2, *P <* 0.001; *Χ*^2^ = 11.8, *P =* 0.008).

It is also worth noting that precarity showed significant main effects on prosociality (Table S5). These associations with precarity differed based on whether the type of prosociality was financial or nonfinancial. Interestingly, across both subjective and income, the experience of precarity increased prosociality for the nonfinancial measures of trust, negative reciprocity, volunteering, and helping a stranger. For the financial measures: altruism, donating money, and positive reciprocity, experiencing precarity was associated with less prosociality.

### Associations between wealth and prosociality are largely consistent across countries

**Fig. 3.**
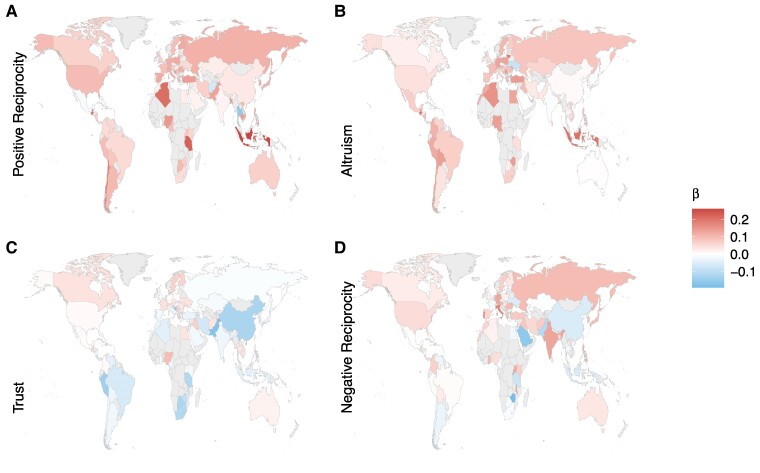
Associations of income with prosocial preferences across the globe. Income was positively associated with A) positive reciprocity in 69/76 (91%, binomial proportion test comparing to 50% *P* < 0.001) countries, B) altruism in 67/76 (88%, *P* < 0.001) countries, C) trust in 33/76 (43%, *P* = 0.900) of countries with 43/76 (57%, *P* = 0.200) showing negative relationships, and neither proportion being statistically significant, D) negative reciprocity in 53/76 (70%, *P* < 0.001) countries. β values are standardized regression coefficients. Income was measured by self-reported HHI, adjusted to achieve purchasing power parity for legitimate comparison between countries (see Methods).

**Fig. 4.**
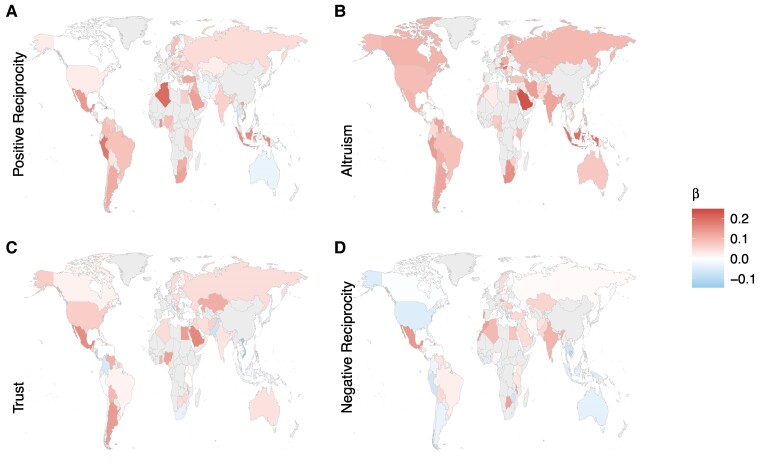
Associations of FWB with prosocial preferences across the globe. FWB was positively associated with A) positive reciprocity in 48/68 (71%, *P* < 0.001) countries, B) altruism in 55/68 (81%, *P* < 0.001) countries, C) trust in 50/68 countries (74%, *P* < 0.001), and D) negative reciprocity in 44/68 countries (65%, *P* = 0.01). β values are standardized regression coefficients. FWB was measured by a four-item scale capturing participants’ perceptions of their personal economic situation (see Methods).

**Fig. 5.**
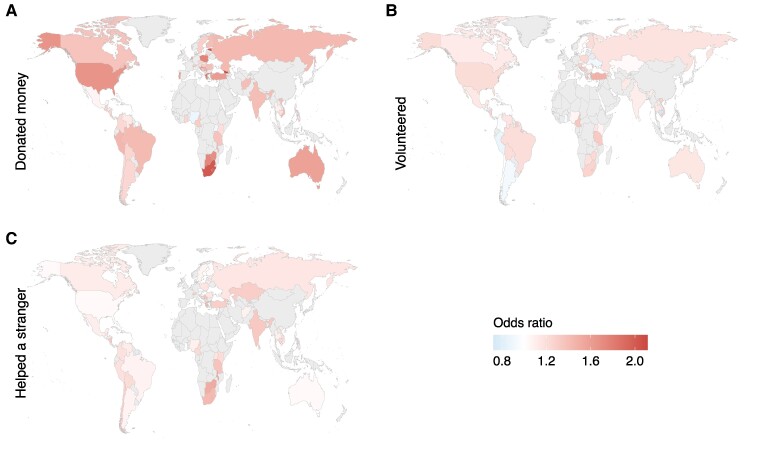
Associations of income with prosocial behaviors across the globe. Income had a positive association with A) donating in 52/57 (91%, *P* < 0.001) countries, B) volunteering in 45/57 countries (79%, *P* < 0.001), and C) helping a stranger in 48/57 countries (84%, *P* < 0.001). ORs > 1 mean the behavior is more likely with increased income representing a positive association, and vice versa for OR < 1.

**Fig. 6.**
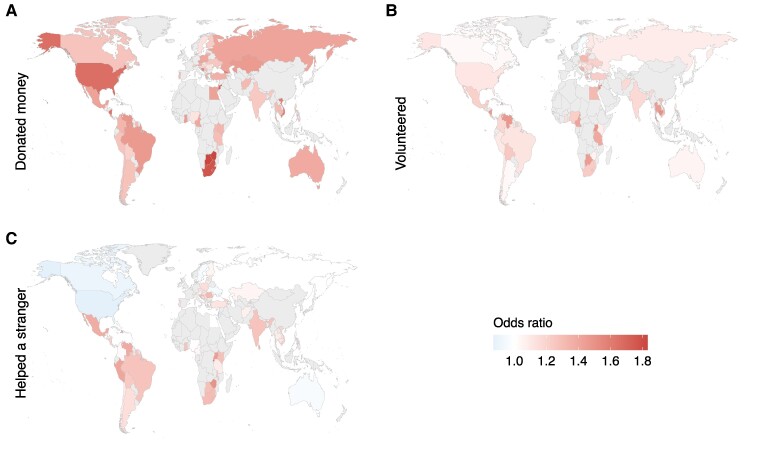
Associations of FWB on prosocial behaviors across the globe. FWB had positive relationships with A) donating in every country measured 59/59 (100%, *P* < 0.001), B) volunteering in 57/59 (97%, *P* < 0.001) countries, and C) helping a stranger in 49/59 countries (83%, *P* < 0.001). ORs > 1 mean the behavior is more likely with increased income representing a positive association, and vice versa for OR < 1.

Having demonstrated that increased wealth is associated with increased prosociality, we next examined the consistency of associations across countries. Previous studies have generally focused on Western, Educated, Industrialized, Rich, and Democratic (“WEIRD”) samples (7, 17, 24, 39–42), and therefore, whether associations between wealth and prosociality vary around the world is unclear. We fitted (G)LMs with fixed effects of wealth and the control variables, to the data for each country separately (see Methods), and extracted the standardized effect sizes, plotted on global maps, regardless of significance (Figs. 3–6). We also calculated two simple statistics, the proportion of countries matching the direction of the global wealth correlation (positive or negative) and the proportion of countries that showed statistically significant effects (*P* < 0.05), either with or contrary to the global pattern (Table S6). As a measure of global consistency, we tested whether a significant (*P* < 0.05) majority of countries showed the same wealth association as the overall sample with a binomial proportion test (H_0_: proportion *=* 0.5).

Associations with both income and FWB were highly consistent around the world. A positive correlation with wealth was found in the majority of countries for positive reciprocity (income = 91%, *P <* 0.001; FWB = 82%, *P <* 0.001), altruism (income = 88%, *P <* 0.001; FWB = 94%, *P <* 0.001), negative reciprocity (income = 70%, *P <* 0.001; FWB = 65%, *P =* 0.01), donating money (income = 91%, *P* < 0.001; FWB = 100%, *P <* 0.001), volunteering (income = 79%, *P* < 0.001; FWB = 97%, *P <* 0.001), and helping a stranger (income = 84%, *P* < 0.001; FWB = 83%, *P <* 0.001). Interestingly, trust showed a more even split with 57% of countries showing a negative correlation of income (*P* = 0.200), as in the overall model, but 43% of countries showing a positive correlation (*P* = 0.900), and neither proportion was significantly different from 50%. When modeled against FWB, trust showed a consistent positive pattern (74%, *P <* 0.001).

### Moderation of associations by country-level GNI and cultural dimensions

While wealth was positively associated with prosociality across most countries, our final analysis examined three potential country-level moderators—per capita GNI (preregistered), the strength of family relationships (preregistered), and cultural individualism–collectivism (exploratory). This analysis measured if our correlations were robust to country-level factors serving as an additional control analysis for global consistency of relationships. Family Ties measures the importance of intra-family relationships and is correlated with social and economic outcomes including trust, labor market participation, stress, and well-being (43). We also explored the moderating influence of individualism–collectivism (44) as prosocial norms can vary by culture (45). Correlations with the other country-level moderators showed stronger Family Ties was associated with collectivism (*r*_(25)_=−0.50, *P* = 0.007) and higher GNI with individualism (*r*_(41)_=0.76, *P* < 0.001).

**Fig. 7.**
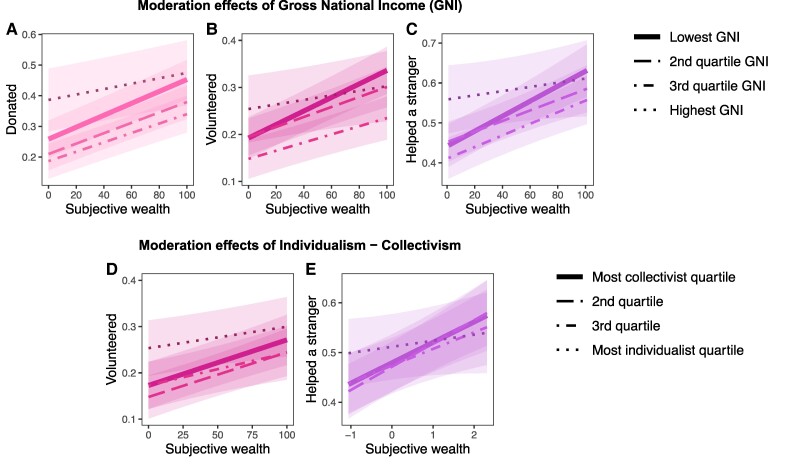
Moderation of GNI and individualism–collectivism on the wealth–prosociality association by quartile. GNI moderated the association of FWB with A) donating, B) volunteering, and C) helping a stranger. Positive wealth correlations were smaller in the richest countries but remained consistently positive in all quartiles. Individualism–collectivism moderated D) volunteering and E) helping a stranger. The association between wealth and prosociality was smaller in more individualistic countries, but again remained positive across quartiles.

GNI did not significantly moderate any wealth associations with prosocial preferences (Table S7). However, GNI did significantly moderate FWB associations with the behaviors: donating (OR = 0.94 [0.90, 0.98], *P =* 0.005), volunteering (OR = 0.94 [0.91, 0.98], *P =* 0.001), and helping a stranger (OR = 0.94 [0.90, 0.98], *P =* 0.001). To understand these interactions, we split the data into quartiles. Countries with the highest GNI had smaller wealth associations, due to high levels of prosociality among the relatively less wealthy people in the richest countries (Fig. 7A–C). That the association between wealth and prosociality remained positive in all country-level income bands further supports remarkable consistency around the world.

For Family Ties, there was no significant moderation on any of the prosocial preferences or behaviors (Table S9). As with GNI, there were no significant interactions between individualism–collectivism and wealth with preferences (Table S8). In contrast, in more collectivist cultures the positive relationship with FWB was significantly stronger for helping a stranger (OR = 0.94 [0.90, 0.98], *P* < 0.001) and volunteering (0.95 [0.91, 0.98], *P* < 0.001; Fig. 7D and E), but it remained consistently positive in individualist cultures. Overall, although country-level factors moderated wealth–prosociality associations, associations were consistently positive.

## Discussion

Prosociality is essential for the well-being of individuals, communities, and society as a whole (5, 6, 9). We found that wealth, which we employ as a general term representing both self-reported income and subjective FWB, was positively associated with most measures of self-reported prosociality, consistently across the world. Income was positively associated with altruism, positive reciprocity, negative reciprocity (a tendency to punish, important to maintain co-operative behavior), donating money, volunteering, and helping a stranger, although negatively associated with trust. FWB was positively associated with all measures, including trust. Moderation analyses demonstrated that experience of precarity, country-level per capita GNI, and individualism–collectivism, explained variance in associations, yet all the wealth–prosocial relationships remained positive.

Our findings significantly extend previous research suggesting a positive association between income and prosociality (9, 11–13, 46, 47). We showed that these associations were present across a wide range of financial and nonfinancial measures of prosociality. Furthermore, we demonstrate high consistency across the world. We also showed moderation by per capita GNI and individualism–collectivism. The association of wealth and prosociality was weaker in countries that were the most individualistic and had the highest GNI, but remained consistently positive.

In contrast to HHI, we hypothesized that FWB would be negatively associated with prosocial preferences and behaviors (16, 21–23, 48). Instead, we found that all prosocial preferences and behaviors were positively associated with FWB, and these results were also consistent globally. There are several possible reasons for these contrasting findings. Firstly, the results of Piff et al. have been cast into doubt based on their methodology and failed replications (20, 26, 27, 29, 30, 49, 50). The size and representativeness of our sample, including hard-to-find groups, may also be a factor when previous studies have used WEIRD samples. Even within studies that recruit from non-WEIRD countries, reliance on online recruitment and testing can bias samples in terms of education and internet access (51).

Secondly, previous studies have often used measures of subjective SES, a self-report regarding how well one thinks one is faring compared with others in their country based on financial status, education, and occupation (52). This measure may be defined differently in different countries contributing to mixed findings (20). People who feel greater FWB report higher prosociality. Associations between income and FWB were modest in our study, supporting measuring these constructs have somewhat independent relationships with prosociality.

In the context of positive associations of both subjective and income on most prosocial measures, the negative association of income and trust is notable. Research has shown that country-level GNI and trust are positively correlated globally (53, 54) and in individual countries (55). Two aspects of our findings on trust are worth considering. Firstly, we showed the importance of including country-level random effects when assessing associations between wealth and trust. Without these, there was a positive global correlation between trust and income, but without them, the relationship was negative.

Secondly, additional variables may explain positive correlations between trust and wealth. Cognitive ability (in the form of literacy skills) is an important factor in the relationship between trust and income (56) and we controlled for cognitive ability in our study. If other studies were not able to control for cognitive ability, they may have overestimated the positive relationship between wealth and trust, which we found to be the case for the studies previously cited (Table S10). In the case where effect sizes are small, this could make the difference between concluding a positive versus a negative association. Future studies could examine the mechanisms that drive reduced trust with increasing wealth.

It is important to highlight that the psychological phenomenon of prosociality carries a strong moral component (57–60). People who think and behave more prosocially may be judged more positively by others (61). But different responses may be nothing to do with someone's moral character, but rather an adaptive response to circumstances (62). Overall, prosociality will be determined by both the desire to help and ability to help. Our findings therefore do not necessarily relate to the personality traits or motivations of people at differing levels of wealth. Indeed, although wealth was positively associated with prosocial preferences and behaviors that were not financial, we also showed, as predicted, that wealth had a larger correlation with financial measures of prosociality—those involving monetary giving—than nonfinancial measures. This is not surprising as people with higher wealth may be able to give more resources.

We also showed a direct relationship of precarity with prosociality as well as moderation of wealth and prosociality associations. The relationship between precarity and wealth differed between financial and nonfinancial forms of prosociality. Those who had experienced precarity showed higher prosociality on nonfinancial measures. In contrast, our main results show that lower wealth was associated with lower prosociality, including on nonfinancial measures. This difference demonstrates that the experience of struggling to afford basic needs is a qualitatively different experience to the feeling of being unsatisfied with ones’ standard of living. In addition, precarity also moderated wealth associations differently between prosocial preferences and behaviors. Experience of precarity amplified the increase in prosocial behaviors with higher wealth, yet reduced the increase in prosocial preferences with higher wealth. Previous research has varied in whether the prosocial measure is financial or nonfinancial, such as returning letters (42), whereas our study includes both to compare. Together, this pattern of results could help to explain some of the conflicting previous findings on whether higher SES is associated with increased or decreased prosocial behavior (20, 26, 27, 29). Measures of SES may capture or confound both precarity and wealth, which are related but distinct concepts. Future work could assess additional moderators that affect the association between wealth and prosociality. For example, whether these associations are modified by beliefs about the worthiness of the recipient (19) is an important open question.

Overall, our results provide clear evidence of the globally representative association between wealth and prosociality, across a range of financial and nonfinancial measures. As a cross-sectional study, it is important to recognize limitations in terms of causal inference. While it is plausible that individual wealth leads to greater prosociality, it is also possible the opposite may occur, such as in the “investment model” of volunteering (63) or the idea of a “prosocial premium” that benefits labor market success (9). Prosociality and wealth could also influence each other bidirectionally. Manipulating wealth experimentally in a global sample is challenging. However, there is some evidence that local direct cash transfers to poorer households may increase trust and gift-giving (64). Future studies could design ways to manipulate wealth experimentally to test causal links between wealth and prosociality (65).

It is also important to note the strengths and limitations of the measures used. There is evidence that multiitem scales breaking down income into different categories are more accurate for assessing total HHI in countries such as the United Kingdom (66). A single-item measure of HHI is used in our data for speed and global applicability. Multiitem income scales would be challenging to implement across different countries that differ in factors such as taxation, benefits, and corruption, whereas HHI is easily applicable and standardized across countries. Our data are also based on self-report which could be affected by demand characteristics. Future research could use experimental or observation measures of prosocial behavior, combined with manipulations of wealth and precarity to test causality and specific hypotheses about potential mechanisms. However, we selected the prosocial preference measures as they are externally validated against behavior in economic games (67). For prosocial behaviors, participants reported if they had engaged in the behavior or not, creating binary measurements of behaviors that are often continuous or complex in the real world, such as charity donations.

Relatedly, two of our prosocial preference measures (altruism and positive reciprocity) involved participants imagining receiving an amount of money, which could be valued differently by individuals of differing wealth levels. Although we showed the same positive wealth–prosociality associations for income and FWB and with nonfinancial measures of prosociality, future studies could consider measures of giving in proportion to income. A further strength of our results was international consistency. This was reinforced by moderation analyses that demonstrated while some associations are significantly moderated by country-level GNI or individualism–collectivism, the wealth–prosociality associations remain positive. This is particularly important as in some countries, such as the United States and United Kingdom, charity donations are tax deductible, meaning that it can be complicated to uncover direct wealth–prosociality associations within charity donation statistics.

## Conclusion

We show that wealth is associated with higher levels of prosociality on financial and nonfinancial measures of prosocial preferences and behaviors. This pattern was highly consistent around the world, across countries that differ in wealth and cultural factors. In addition, experiencing precarity enhances wealth associations with prosocial behaviors but weakens how strongly wealth is associated with prosocial preferences. The clear implication is that greater levels of prosociality are enabled by increased levels of income and FWB, an important consideration for developing social policies.

## Methods

The main measures were taken from the GPS and GWP. The GWP is a globally representative dataset covering 80,337 individuals, drawn as representative samples from 76 countries, representing 90% of both the world's population and global income (68). Individual countries are sampled with 1,000 respondents per country through a combination of face-to-face and telephone data collection. The GPS adds specific questions regarding economic preferences and behaviors (67) to the GWP. Two further data sources were used for the analyses of economic and social moderators. GNI data were taken from the World Bank Open Data (69). The measure of importance of family ties was taken from the World Values Survey (70). Analysis of these secondary datasets was approved by the University of Birmingham Science, Technology, Engineering and Mathematics ethics committee (ERN_20-1897PA), and details of the consent procedures are provided in the original manuscripts for each dataset.

### Wealth measures

We used self-reported HHI, adjusted to achieve purchasing power parity to be comparable across different countries (71), and log-transformed (see Supplementary Methods). FWB is a four-item index from the GWP capturing participants’ feelings about their economic situation (Supplementary Methods).

### Prosociality measures

The GPS measures four prosocial *preferences*: positive reciprocity, altruism, trust, and negative reciprocity, developed through independent experimental validation (67) (Supplementary Methods). The GPS defines positive reciprocity as the propensity to return a prosocial act (two items), altruism as willingness to give to good causes without expecting anything in return (two items), trust as assuming people have good intentions (one item), and negative reciprocity as the tendency to punish unfair behavior (three items; Supplementary Methods). The GWP measures three prosocial *behaviors*: donating money, volunteering, and helping a stranger. Participants reported whether they have done each in the past month, creating binary measures. Some missing data were imputed in the original GPS dataset following the procedures in Appendix AH: https://doi.org/10.1093/qje/qjy013

### Moderators

Individual-level precarity was quantified using the GWP Food and Shelter Index. This measure was only weakly (72) correlated with FWB (Cramer’s *V*_(18, *N* = 66,654)_ = 0.156), suggesting that they could be evaluated independently. Furthermore, 29% of those who reported experiencing precarity were high in FWB, confirming that each FWB category contained people who experienced precarity. Country-level moderators were GNI from the World Bank Open Data (69), Family Ties from the World Values Survey (70), and Individualism–Collectivism (44) (Supplementary Methods).

### Analysis

All analyses were run using R (v4.1.0) in R Studio (v2022.12.0). As preregistered, we tested income and FWB as explanatory variables in (generalized) mixed-effect models (G)LMMs of each measure of prosociality. Control variables, used in all models, were gender, health (GWP Physical Well-Being Index), age, and cognitive ability (GWP self-reported math ability). Income, FWB, and control variables were entered as fixed and random effects grouped by country with a country-level intercept. Random effects of gender caused model convergence issues and were removed. All continuous variables were standardized and mean centered. Significance is reported at *P <* 0.01 for analyses using the full sample.

To assess consistency around the world, we calculated wealth associations in each country with all the controls available for that country. We also calculated (i) the proportion of countries that had the same directional association as globally, regardless of significance, and (ii) the proportion of significant associations (*P <* 0.05) in each direction and expressed these as a proportion of the total number of countries. A one-tailed binomial proportion significance test assessed whether the proportions were significantly above 0.5. Analysis of individual- and country-level moderators repeated each wealth–prosocial model, adding the moderating variable as a direct association and interaction with wealth. For precarity, a four-level factorial variable, interactions were assessed with an omnibus ANOVA.

## Supplementary Material

pgae582_Supplementary_Data

## Data Availability

The GPS survey data are available from the Institute of Labour Economics: https://gps.iza.org/home. GWP survey data are commercially available via Gallup: https://www.gallup.com/analytics/213617/gallup-analytics.aspx. GNI data are available from the World Bank: https://databank.worldbank.org/source/world-development-indicators. All publicly available datasets and study materials used in this study and the preregistration are available from https://osf.io/z35fm/. All analysis scripts are publicly available (https://osf.io/z35fm/).

## Preregistration

The hypotheses, methods, and analysis plan were preregistered (https://aspredicted.org/K1R_KNJ) on 2022 June 20. There was one minor deviation from the preregistration (for details, see main article Results).

## Preprints

This manuscript was posted on a preprint: https://osf.io/z35fm/.
