# Supplementary material for: Greater income and financial well-being are associated with higher prosocial preferences and behaviors across 76 countries
Source: PNAS Nexus. 2025 Feb 4;4(2):pgae582. doi: 10.1093/pnasnexus/pgae582 (PMC11792073; doi:10.1093/pnasnexus/pgae582)
Supplement: pgae582_Supplementary_Data [file pgae582_supplementary_data.pdf]

Supplementary Results

For all tables β represents regression coefficients, OR = odds ratios and CI = confidence intervals.

Table S1: Quadratic effects of all wealth-prosociality models

Wealth measure	Prosociality measure	β (x^2)	CI low	CI high	*p*-value
	Preferences (LMM standardized regression coefficients)				
Subjective	Altruism	-3.97	-5.96	-1.98	**<0.001**
Objective	Altruism	0.39	-2.38	3.15	0.780
Subjective	Negative reciprocity	-1.27	-3.33	0.79	0.230
Objective	Negative reciprocity	1.68	-1.26	4.62	0.260
Subjective	Positive Reciprocity	-0.75	-2.81	1.31	0.470
Objective	Positive Reciprocity	3.03	0.10	5.96	0.043
Subjective	Trust	-5.35	-7.43	-3.27	**<0.001**
Objective	Trust	-3.36	-6.13	-0.58	0.018
	Behaviours (GLMM odds ratios)				
Subjective	Donated	-16.43	-21.58	-11.28	**<0.001**
Objective	Donated	12.78	4.79	20.76	**0.002**
Subjective	Volunteered	-8.24	-13.65	-2.84	**0.003**
Objective	Volunteered	9.34	2.30	16.38	**0.009**
Subjective	Helped	-9.73	-14.37	-5.09	**<0.001**
Objective	Helped	4.17	-2.43	10.77	0.220

Note: the effects reported here are orthogonal to the lower order main effects, and as a result the coefficients are not directly comparable. This is done so that independent significance of quadratic effects in the models can be seen.

Table S2: Main effects of all wealth-prosociality models

Wealth measure	Prosociality measure	Main effect	CI low	CI high	*p*-value
	Preferences (LMM standardized regression coefficients)				
Subjective	Altruism	0.085	0.072	0.098	**<.001**
Objective	Altruism	0.097	0.077	0.117	**<.001**
Subjective	Negative reciprocity	0.025	0.009	0.041	**0.002**
Objective	Negative reciprocity	0.051	0.027	0.075	**<.001**
Subjective	Positive Reciprocity	0.065	0.050	0.081	**<.001**
Objective	Positive Reciprocity	0.112	0.089	0.136	**<.001**
Subjective	Trust	0.033	0.018	0.049	**<.001**
Objective	Trust	-0.027	-0.046	-0.008	**0.005**
	Behaviours (GLMM odds ratios)				
Subjective	Donated	1.384	1.334	1.436	**<.001**
Objective	Donated	1.562	1.443	1.690	**<.001**
Subjective	Volunteered	1.221	1.177	1.267	**<.001**
Objective	Volunteered	1.157	1.099	1.218	**<.001**
Subjective	Helped	1.200	1.153	1.250	**<.001**
Objective	Helped	1.271	1.210	1.336	**<.001**

Note: significant effects (p<.01) in bold

Table S3: Comparison of regression coefficients from main models using z-score

Comparison pair	β_1 / OR_1	β_2 / OR_2	z-score	*p*-value
OBJECTIVE WEALTH - PREFERENCE MODELS				
Altruism - Trust	0.097	-.027	12.25	**< .001**
Altruism – Positive reciprocity	0.097	0.112	-0.98	.167
Altruism – Negative reciprocity	0.097	0.051	4.07	**< .001**
Trust – Positive reciprocity	-.027	0.112	-11.07	**< .001**
Trust – Negative reciprocity	-.027	0.051	-12.48	**< .001**
Positive reciprocity – negative reciprocity	0.112	0.051	4.53	**< .001**
OBJECTIVE WEALTH - BEHAVIOUR MODELS				
Donating – Volunteering	0.446	0.146	3.24	**< .001**
Donating – Helping	0.446	0.240	2.11	.017
Volunteering - Helping	0.146	0.240	-2.10	.018
SUBJECTIVE WEALTH - PREFERENCE MODELS				
Altruism – Trust	0.085	0.033	6.90	**< .001**
Altruism – Positive reciprocity	0.085	0.065	2.21	.014
Altruism – Negative reciprocity	0.085	0.025	8.26	**< .001**
Trust – Positive reciprocity	0.033	0.065	-4.93	**< .001**
Trust – Negative reciprocity	0.033	0.025	2.20	.014
Positive reciprocity – negative reciprocity	0.065	0.025	6.48	**< .001**
SUBJECTIVE WEALTH - BEHAVIOUR MODELS				
Donating – Volunteering	0.325	0.200	2.38	**.009**
Donating – Helping	0.325	0.182	2.75	**.003**
Volunteering - Helping	0.200	0.182	0.47	.320

Note: significant effects (p<.01) in bold

Table S4: Model comparison results of precarity as a moderator on the wealth-prosociality association

	Objective wealth			Subjective wealth		
	X^2	*p*-value	Direction	X^2	*p*-value	Direction
			Preferences			
Positive reciprocity	8.6	0.036	-	**31.5**	**<.001**	**Negative**
Altruism	**15.1**	**0.002**	**Negative**	3.7	0.297	-
Trust	5.4	0.144	-	0.1	0.992	-
Negative reciprocity	1.2	0.756	-	4.9	0.176	-
			Behaviours			
Donating	5.5	0.136	-	**19.0**	**<.001**	**Positive**
Volunteering	1.2	0.762	-	**15.4**	**0.002**	**Positive**
Helping	**21.2**	**<.001**	**Positive**	**11.8**	**0.008**	**Positive**

Note: significant effects (p<.01) in bold. X^2 is chi-square statistic.

Table S5: Main effects of precarity on prosocial preferences and behaviours controlling for wealth (showing only effects where *p*<.01)

	Precarity measure	Wealth control	Main effect coefficient	CI Low	CI High	*p*-value
			β			
Altruism	F/S	Objective	-0.0629	-0.0887	-0.0371	<0.001
	F/S	Subjective	-0.0696	-0.0939	-0.0452	<0.001
Positive reciprocity	F	Objective	-0.0343	-0.0597	-0.0089	0.008
	F/S	Objective	-0.0515	-0.0776	-0.0254	<0.001
	F	Subjective	-0.0387	-0.0645	-0.0129	0.003
	F/S	Subjective	-0.0707	-0.0960	-0.0455	<0.001
Negative reciprocity	F/S	Objective	0.0636	0.0372	0.0900	<0.001
	F/S	Subjective	0.0630	0.0376	0.0883	<0.001
Trust	F/S	Objective	0.0510	0.0246	0.0774	<0.001
	F/S	Subjective	0.0781	0.0525	0.1036	<0.001
			log-odds			
Donating	F	Objective	-0.1196	-0.1954	-0.0439	0.002
Volunteering	S	Objective	0.1830	0.0780	0.2880	<0.001
	F/S	Objective	0.2215	0.1412	0.3019	<0.001
	S	Subjective	0.2221	0.1177	0.3265	<0.001
	F/S	Subjective	0.2700	0.1993	0.3407	<0.001
Helping a stranger	F	Objective	0.1254	0.0573	0.1935	<0.001
	S	Objective	0.1286	0.0382	0.2191	0.005
	F/S	Objective	0.1266	0.0565	0.1968	<0.001
	F	Subjective	0.1300	0.0654	0.1946	<0.001
	S	Subjective	0.1337	0.0429	0.2246	0.004

Note: F = Food precarity (in the last 12 months), S = Shelter, F/S = Food and Shelter

Table S6: Per country model effect sizes

Effect	Proportion of positive country effects	Proportion of negative country effects	Binomial test (H_0 = .50; H_1 > H_0) p-value	Proportion of positive effects significant (*p*<.05)	Proportion of negative effects significant (*p*<.05)
Altruism	67/76 **(88%)**	9/76 (12%)	**< .001**	36/67 (54%)	1/9 (11%)
Positive reciprocity	69/76 **(91%)**	7/76 (9%)	**<.001**	41/69 (59%)	1/7 (14%)
Negative reciprocity	53/76 **(70%)**	23/76 (30%)	**<.001**	23/53 (43%)	6/23 (26%)
Trust	33/76 (43%)	43/76 **(57%)**	.900	2/33 (6%)	12/43 (28%)
Donating	52/57 **(91%)**	5/57 (9%)	**< .001**	45/52 (87%)	0/5 (0%)
Volunteering	45/57 **(79%)**	12/57 (21%)	**< .001**	11/44 (24%)	1/12 (8%)
Helping	48/57 **(84%)**	9/57 (16%)	**< .001**	26/48 (54%)	0/9 (0%)
Altruism	64/68 **(94%)**	4/68 (6%)	**< .001**	45/64 (70%)	0/4 (0%)
Positive reciprocity	56/68 **(82%)**	12/68 (18%)	**< .001**	30/56 (54%)	0/12 (0%)
Negative reciprocity	44/68 **(65%)**	24/68 (35%)	**.010**	12/44 (27%)	6/24 (25%)
Trust	50/68 **(74%)**	18/68 (26%)	**<.001**	18/50 (36%)	4/18 (22%)
Donating	59/59 **(100%)**	0/59 (0%)	**< .001**	52/59 (88%)	0/0 -
Volunteering	57/59 **(97%)**	2/59 (3%)	**< .001**	29/57 (51%)	0/2 (0%)
Helping	49/59 **(83%)**	10/59 (17%)	**< .001**	30/49 (61%)	0/10 (0%)

Note: Columns show numbers of countries matching the criteria with proportions in parentheses. The proportions shown in **bold** match the direction of the global effect. The third column shows whether those proportions were significant at *p*<.01. The final two columns show the proportion of each effect direction that were found to be significant at *p*<.01 for a main effect.

Table S7: Interaction effects between country GNI and wealth

Wealth measure	Prosociality measure	Interaction coefficient	95% CI	*p*-value
		β		
Subjective	Altruism	0.00	(-0.01, 0.02)	0.708
Objective	Altruism	0.00	(-0.02, 0.02)	0.891
Subjective	Negative reciprocity	0.00	(-0.02, 0.02)	0.795
Objective	Negative reciprocity	0.03	(0.00, 0.05)	0.043
Subjective	Positive Reciprocity	-0.01	(-0.03, 0.01)	0.231
Objective	Positive Reciprocity	0.01	(-0.15, 0.01)	0.427
Subjective	Trust	0.00	(-0.02, 0.02)	0.938
Objective	Trust	0.02	(0.00, 0.04)	0.049
		log-odds		
Subjective	Donated	-0.06	(-0.10, -0.02)	**0.005**
Objective	Donated	0.10	(0.01, 0.19)	0.036
Subjective	Volunteered	-0.06	(-0.09, -0.02)	**0.001**
Objective	Volunteered	-0.02	(-0.09, 0.06)	0.695
Subjective	Helped	-0.06	(-0.10, -0.02)	**0.001**
Objective	Helped	-0.05	(-0.10, -0.00)	0.060

Note: significant effects (*p*<.01) in bold

Table S8: Interaction effects between Individualism-Collectivism and wealth

Wealth measure	Prosociality measure	Interaction coefficient	95% CI	*p*-value
		β		
Subjective	Altruism	0.00	(-0.01, 0.02)	0.682
Objective	Altruism	0.00	(-0.03, 0.02)	0.657
Subjective	Negative reciprocity	0.00	(-0.03, 0.03)	0.921
Objective	Negative reciprocity	0.03	(0.00, 0.06)	0.048
Subjective	Positive Reciprocity	-0.02	(-0.04, -0.00)	0.042
Objective	Positive Reciprocity	0.00	(-0.03, 0.03)	0.964
Subjective	Trust	0.00	(-0.02, 0.02)	0.785
Objective	Trust	0.01	(-0.00, 0.03)	0.232
		log-odds		
Subjective	Donated	-0.03	(-0.07, 0.00)	0.110
Objective	Donated	0.10	(0.01, 0.18)	0.022
Subjective	Volunteered	-0.05	(-0.09, -0.02)	**< .001**
Objective	Volunteered	0.03	(-0.03, 0.08)	0.367
Subjective	Helped	-0.06	(-0.10, -0.02)	**0.005**
Objective	Helped	-0.04	(-0.10, 0.02)	0.168

Note: significant effects (*p*<.01) in bold

Table S9: Interaction effects between Family Ties and wealth

Wealth measure	Prosociality measure	Interaction coefficient	95% CI	*p*-value
		β		
Subjective	Altruism	-0.01	(-0.04, 0.01)	0.178
Objective	Altruism	0.00	(-0.02, 0.03)	0.817
Subjective	Negative reciprocity	0.00	(-0.03, 0.03)	0.921
Objective	Negative reciprocity	-0.01	(-0.05, 0.02)	0.425
Subjective	Positive Reciprocity	-0.03	(-0.06, -0.01)	0.023
Objective	Positive Reciprocity	0.01	(-0.02, 0.05)	0.374
Subjective	Trust	0.00	(-0.02, 0.02)	0.960
Objective	Trust	0.00	(-0.03, 0.02)	0.667
		log-odds		
Subjective	Donated	0.00	(-0.05, 0.06)	0.842
Objective	Donated	0.08	(-0.08, 0.25)	0.331
Subjective	Volunteered	-0.02	(-0.07, 0.03)	0.400
Objective	Volunteered	0.00	(-0.08, 0.08)	0.974
Subjective	Helped	0.00	(-0.08, 0.06)	0.798
Objective	Helped	0.06	(0.00, 0.12)	0.048

Note: significant effects (*p*<.01) in bold

Table S10: Cited papers for income-wealth relationships showing cognitive ability controls

Paper	Cognitive ability controls
(Ananyev & Guriev, 2019)	Educational level included, but none for cognitive ability
(Steijn & Lancee, 2011)	Educational level included, but none for cognitive ability
(Morrone, 2009)	None
(Brandt et al., 2015)	None

Table S11: Variance Inflation Factors for GNI x Household Income

Wealth measure	Prosociality measure	VIF GNI main effect	VIF GNI interaction effect
Objective	Altruism	1.014	1.010
Objective	Negative reciprocity	1.017	1.012
Objective	Positive Reciprocity	1.014	1.010
Objective	Trust	1.027	1.018
Objective	Donated	1.520	1.519
Objective	Volunteered	3.641	3.595
Objective	Helped	3.956	3.878

Fig. S1: Results of linear mixed models for prosocial preferences and objective wealth

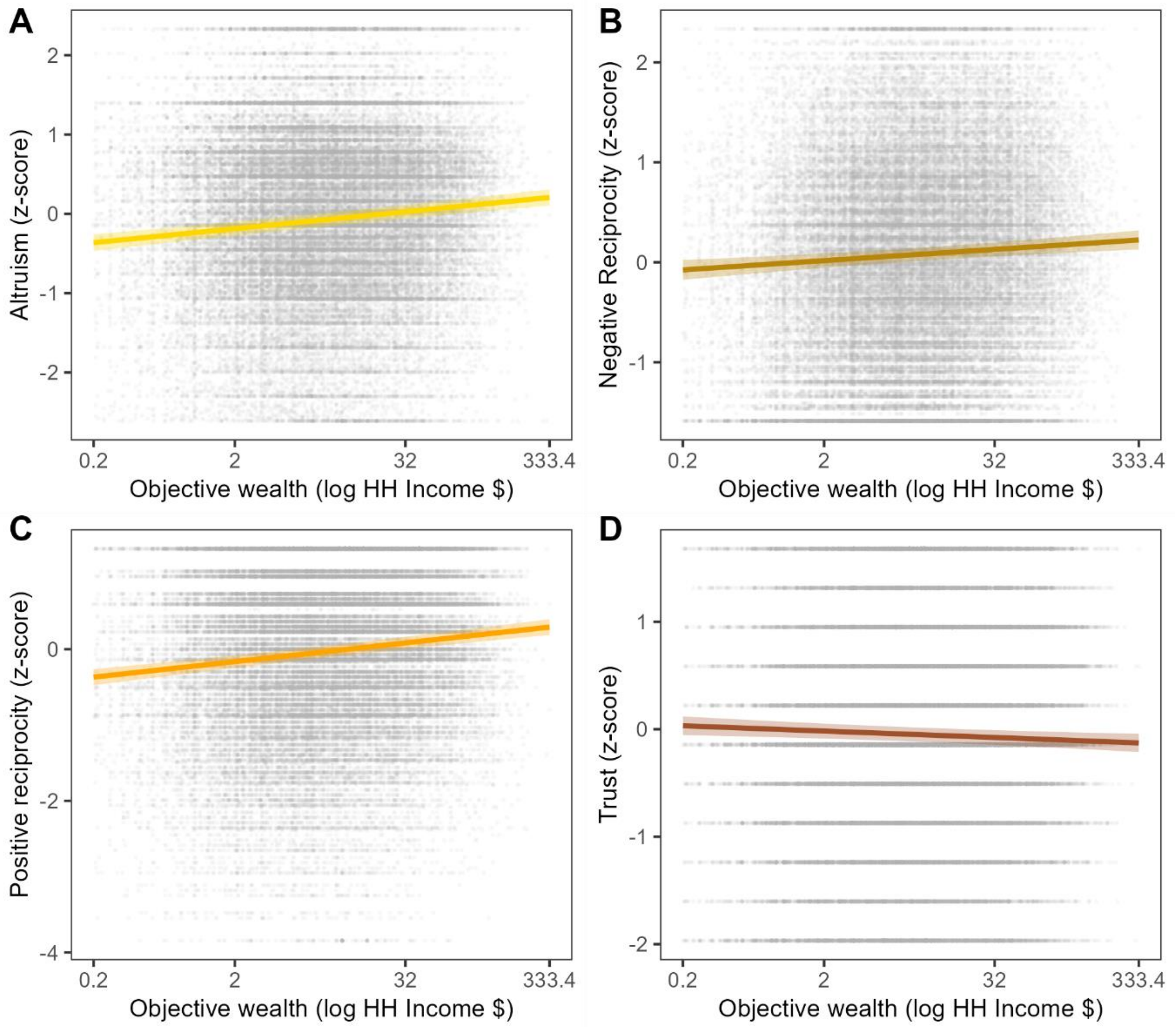

Fig S1 | Results of linear mixed models for prosocial preferences and objective wealth. Objective wealth showed positive associations with **(A)** altruism**, (B)** negative reciprocity and **(C)** positive reciprocity, and a negative association with **(D)** trust. Plots show predictions from linear mixed-effect models (LMMs) of prosocial preferences / behaviours, controlling for gender, age, physical health and cognitive ability. Preferences were modelled as standardised continuous variables and binary behaviours were modelled with generalised LMMs (see Methods). Linear effects are shown here with the quadratic effects reported in supplementary materials. Plots were created by predicting response data from model fits, the shaded area representing 95% confidence interval. Plots show individual data points for each respondent.

Fig. S2: Results of linear mixed models for prosocial behaviours and objective wealth

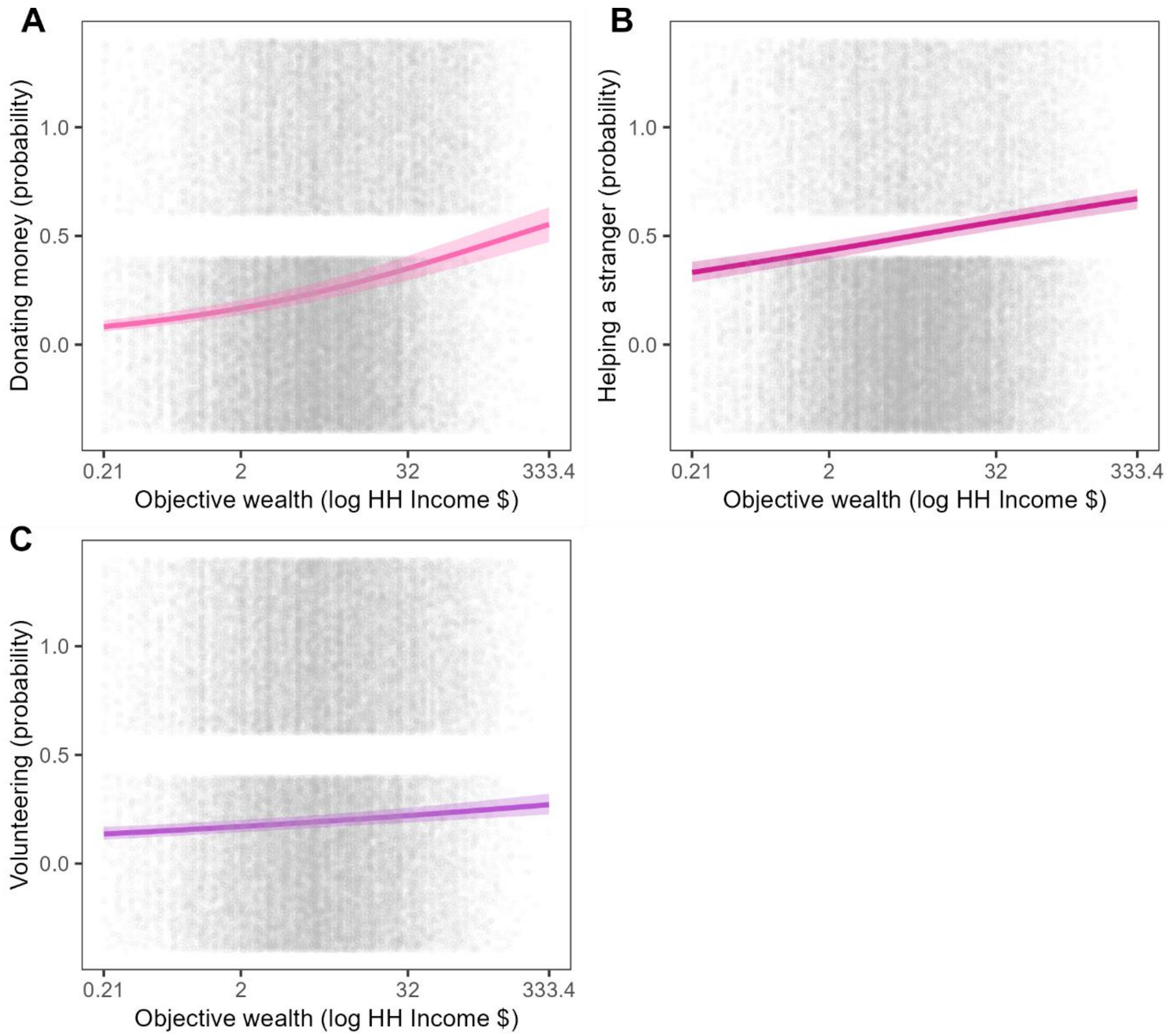

Fig S2 | Results of linear mixed models for prosocial behaviours and objective wealth. Objective wealth showed positive associations with **(A)** donating**, (B)** helping a stranger and **(C)** volunteering. Plots show individual data points for each respondent.

Fig. S3: Results of linear mixed models for prosocial preferences and subjective wealth

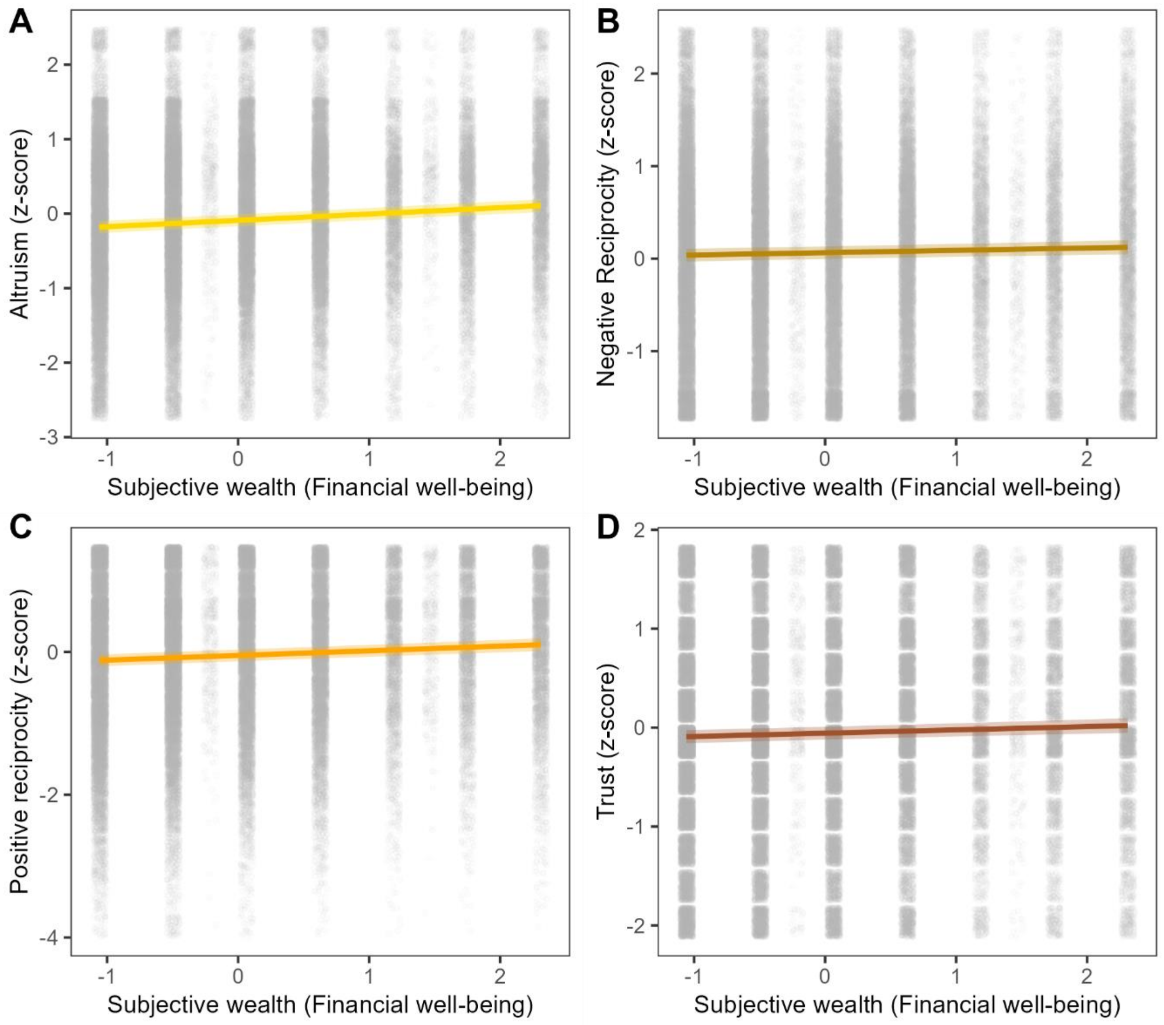

Fig S3 | Results of linear mixed models for prosocial preferences and subjective wealth. Objective wealth showed positive associations with **(A)** altruism**, (B)** negative reciprocity, **(C)** positive reciprocity, and **(D)** trust. Plots show individual data points for each respondent.

Fig. S4: Results of linear mixed models for prosocial behaviours and objective wealth

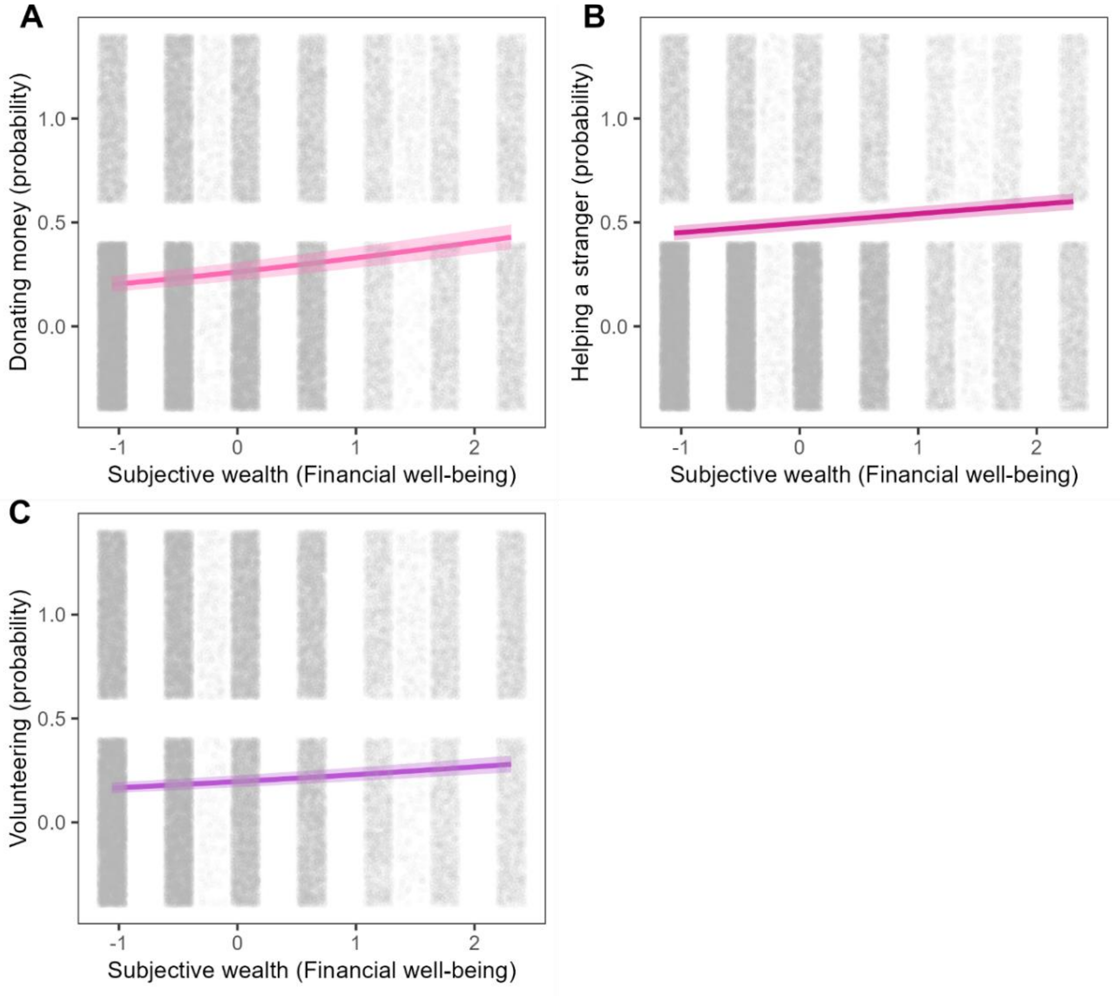

Fig S4 | Results of linear mixed models for prosocial behaviours and subjective wealth. Objective wealth showed positive associations with **(A)** donating**, (B)** helping a stranger and **(C)** volunteering. Plots show individual data points for each respondent.

Supplementary Methods

Italicised text taken directly from Gallup World Poll methodology, September 2012 (Gallup, Inc, n.d.)

Wealth measures

Subjective wealth measure

"The Financial Wellbeing Index measures respondents' personal economic situations and the economics of the community where they live."

Index Questions

"Which one of these phrases comes closest to your own feelings about your household's income these days: living comfortably on present income, getting by on present income, finding it difficult on present income, or finding it very difficult on present income?"
"Are you satisfied or dissatisfied with your standard of living, all the things you can buy and do?"
"Right now, do you feel your standard of living is getting better or getting worse?"
"Right now, do you think that economic conditions in the city or area where you live, as a whole, are getting better or getting worse?"

Index Construction

"Index scores are calculated at the individual record level. For each individual record, respondents who say they are "living comfortably on present income" are recoded as "1," and all other answers are recoded as "0." The remaining items are recoded so that positive answers are scored as a "1" and all other answers (including don't know and refused) are assigned a score of "0." A record's final index score is the average of the mean for responses to the first question and the mean of the three other items multiplied by 100."

In line with Gallup's standard procedures, for the first question respondents who said they were "living comfortably on present income" were scored as 1 and all other answers as 0. The remaining three items were scored with positive answers as a 1 and all other answers as 0. The final index score was calculated using the average of the mean for responses to question (i) and the mean of the three other items multiplied by 100.

On examining the data, it was observed that household income had a typical Lorenz curve distribution, with extreme outliers at high incomes and was best modelled using a transformed logarithmic scale yielding an approximately normal distribution in line with other studies (Kosse & Tincani, 2020). This was then standardised, and the resultant z-score used as the predictor variable. As a result of this transformation, quadratic effects were difficult to interpret and only linear relationships are reported in the main results section, with quadratic effects included for completeness (Table S1). In addition to the high-income outliers, there were also a notable number of very low incomes, suggestive of measurement error. Thus, we cropped the log income scale at +/- 3 standard deviations from the mean, resulting in 143 observations (0.2%)

being removed from the analysis. Of these 133 were smaller than -3 SD i.e. less than $199 per annum total household income, and 10 were larger than +3 SD, i.e. greater than $382,825.

Prosociality measures

The GPS defines positive reciprocity as the propensity to return a prosocial act, measured by two items (i) the level of financial gift (€5-30 equivalent) that respondents would give as a gift after a stranger gives them directions, and (ii) self-report Likert scale response 0-11 on how willing they are in general to return favours, these items receiving roughly equal weights. Altruism was defined as (i) self-report Likert scale response 0-11 how willing they respondents would be to give to good causes without expecting anything in return, and (ii) how much of an unexpected €1,000 windfall they would donate to charity. These two items were weighted about equally. Trust was measured by a single item Likert scale 0-10 response on whether respondents assume that other people only have the best intentions (Likert scale, 0-10). Trust in this instance therefore represents social, rather than institutional, trust. Negative reciprocity is the tendency to punish others for unfair behaviour and was measured by three items, (i) Likert scale response 0-10 on how willing respondents were to take revenge if treated unjustly, even if it there is a cost to doing so, (ii) willingness to punish someone for unfair behaviour, either towards themselves or, (iii) towards a third person (prosocial punishment). Each of the three items received roughly equal weighting.

These measures were defined prior to the data collection for the GPS, via the process of an independent external experimental validation. In this process, respondents completed multiple survey items along with incentivised behavioural tasks. The items which correlated most strongly (highest R^2) with actual behaviour in the tasks was then selected for use in the GPS. The behavioural experiment for altruism was measured using amounts donated to a charity in a dictator game. Trust was based on the amounts sent as first moved in two investment games. Similarly, positive reciprocity was measured as the amounts returned as receiver in two investment games. Negative reciprocity was measured by the amount given to punishment after an opponent's defection in a prisoner's dilemma game, and by the minimum offer deemed acceptable in an ultimatum game.

The GWP (which is linked to the GPS at an individual level) provides three measures of prosocial *behaviour*; donating money, volunteering, or helping a stranger. In each case respondents were asked "Have you done any of the following in the past month? How about (i) "donated money to a charity?", (ii) "volunteered your time to an organization?", and (iii) "helped a stranger or someone you didn't know who needed help?". Each item had a simple binary response such that positive answers were scored as a 1 and all other answers given a score of 0. All prosociality measures from the GWP and GPS were rescaled to standardised scores prior to analysis.

Moderators

Precarity (Food & Shelter Index) measure:

"The Food and Shelter Index measures whether a respondent has experienced deprivation in the areas of food and shelter. Two items that ask about respondents' ability to afford food or shelter in the past year compose this index. Lower scores on this index indicate that more

respondents reported struggling to afford food and shelter in the past year, while higher scores indicate fewer respondents reported such struggles."

Index Questions

"Have there been times in the past 12 months when you did not have enough money to buy food that you or your family needed?"
"Have there been times in the past 12 months when you did not have enough money to provide adequate shelter or housing for you and your family?"

Index Construction

"Index scores are calculated at the individual record level. For each individual record the two items are recoded so that positive (or favorable) answers are scored a "1" and all other answers (including don't know and refused) are assigned a score of "0." An individual record has an index calculated if it has valid scores for both questions. A record's final index score is the mean of valid items multiplied by 100."

This index was modelled as a discrete variable with four levels: no precarity, food precarity only, shelter precarity only, and both, testing for fixed effects and their interaction effect with wealth. We used ANOVA omnibus tests to identify for each model whether there was a significant interaction effect overall with the wealth variable (Table S4). For each significant interaction we then conducted post-hoc significance tests using (G)LMM's to establish which specific factor levels were responsible for the differences (Table S5).

We also examined whether three distinct country-level factors moderated associations between income / financial well-being and prosociality by including their fixed effects and the interaction effect with wealth.

Gross National Income measure

For this we used per capita Gross National Income from the World Bank (*GNI per capita, Atlas method (current US$)*, n.d.) (pre-registered).

Family Ties measure

The Family Ties measure (Alesina & Giuliano, 2014) is constructed from the World Values Survey. It measures the strength of relationships within family units at intra- and inter-country levels. The measure is correlated with a range of highly relevant social and economic outcomes; trust, labour market participation, stress, and well-being suggesting good external validity. On obtaining the data it was found that the Family Ties metric as described in in the pre-registration was not available due to a change in the WVS questionnaire for Wave 6 (2010-2014). Instead, the weighted PCA measure was substituted with a single-item response to the question 'How important is family in your life' scaled; 1=Very important, 2=Rather important, 3=Not very important, 4=Not at all important (reverse scored in the analysis) that was available within the same time period of the GPS data.

Individualist-Collectivist culture measure

The individualism-collectivism dimension (Minkov et al., 2017) uses data taken from over 50,000 respondents across 56 countries.

We ran additional models including GNI and family ties respectively as country-level predictors, testing for potential interaction effects with wealth prosociality (Table S7-8). As GNI and HH Income were expected to be correlated we tested models which included both with the variance inflation factor (VIF) to check for collinearity issues. Though there are no strict rule for what constitutes a problematic VIF measure, values of 1 represent complete orthogonality, 5-10 are generally considered cautionary, whereas 10+ is regarded as problematic (Hair, 1992; Thompson et al., 2017). These were found not to be of material concern in our case, with the maximum being 3.96 in behavioural models, and all close to 1 in the preference models (Table S11).

Supplementary References

Alesina, A., & Giuliano, P. (2014). Chapter 4 - Family Ties. In P. Aghion & S. N. Durlauf (Eds.), *Handbook of Economic Growth* (Vol. 2, pp. 177–215). Elsevier.

Ananyev, M., & Guriev, S. (2019). Effect of income on trust: Evidence from the 2009 economic crisis in Russia. *Economic Journal* , *129*(619), 1082–1118.

Brandt, M. J., Wetherell, G., & Henry, P. J. (2015). Changes in income predict change in social trust: A longitudinal analysis. *Political Psychology*, *36*(6), 761–768.

Gallup, Inc. (n.d.). *How Does the Gallup World Poll Work?* Retrieved 17 December 2021, from https://www.gallup.com/178667/gallup-world-poll-work.aspx

GNI per capita, Atlas method (current US$). (n.d.). Retrieved 13 July 2022, from https://data.worldbank.org/indicator/NY.GNP.PCAP.CD

Hair, J. F. (1992). *Multivariate data analysis : with readings*. Macmillan ; Maxwell Macmillan Canada ; Maxwell Macmillan International.

Kosse, F., & Tincani, M. M. (2020). Prosociality predicts labor market success around the world. *Nature Communications*, *11*(1), 5298.

Minkov, M., Dutt, P., Schachner, M., Morales, O., Sanchez, C., Jandosova, J., Khassenbekov, Y., & Mudd, B. (2017). A revision of Hofstede's individualism-collectivism dimension: A new national index from a 56-country study. *Cross Cultural & Strategic Management*, *24*(3), 386–404.

Morrone, A. (2009). *How Good is Trust?* (OECD Statistics Working Papers). Organisation for Economic Co-Operation and Development (OECD). https://doi.org/10.1787/220633873086

Steijn, S., & Lancee, B. (2011). *GINI DP 20: Does Income Inequality Negatively Affect General Trust? Examining three potential problems with the inequality-trust hypothesis*. https://core.ac.uk/outputs/6273040

Thompson, C. G., Kim, R. S., Aloe, A. M., & Becker, B. J. (2017). Extracting the Variance Inflation Factor and Other Multicollinearity Diagnostics from Typical Regression Results. *Basic and Applied Social Psychology*, *39*(2), 81–90.
